# Loose Bodies Found in the Human Intra-Articular Space Showed Characteristics Similar to Endochondral Bone Formation

**DOI:** 10.1177/19476035231212608

**Published:** 2023-12-01

**Authors:** Andrea Schwab, Thomas Pap, Veit Krenn, Wolfgang Rüther, Christoph Lohmann, Jessica Bertrand

**Affiliations:** 1 Department of Orthopaedics, Medical Faculty, Otto-von-Guericke University, Magdeburg, Germany; 2 Department of Orthopaedics and Sports Medicine, Erasmus University Medical Center, Rotterdam, The Netherlands; 3 Institute of Musculoskeletal Medicine, Medical Faculty, Westphalian Wilhelm University, Münster, Germany; 4 MVZ-Zentrum für Histologie, Zytologie und Molekulare Diagnostik GmbH, Trier, Germany; 5 Department of Orthopedics, University Medical Center Hamburg-Eppendorf, Hamburg, Germany

**Keywords:** loose bodies, endochondral ossification, cartilage, osteochondral, osteoarthritis

## Abstract

**Objective:**

Loose bodies are free-floating tissues of cartilage and bone that can cause pain, swelling, the inability to straighten the knee, or intermittent locking of the knee. Loose bodies can arise from degenerative joint disease, flake fractures, osteochondritis dissecans, or chondromatosis. We hypothesized that loose bodies can be classified in stages with tissue characteristics similar to endochondral ossification.

**Design:**

Loose bodies were harvested from patients undergoing joint replacement. Samples were processed for histology, gene expression analysis, and micro-computed tomography (µCT). Cartilage- and bone-related genes and proteins were selected for immunofluorescence stainings (collagen type I, II, and X, SOX9 [SRY-box transcription factor 9], and MMP13 [matrix metalloproteinase 13]) and gene expression analysis (*FN* [fibronectin], *COL1A1, COL2A1, COL10A1, SOX9, MMP13*, and aggrecan [*ACAN*]).

**Results:**

Loose bodies were grouped in 4 stages: fibrous, (mineralized) cartilaginous, cartilage and bone, and bone. Hyaline-like cartilage tissue with Benninghoff arcades was present in stages 2 and 3. A transition from cartilaginous to mineralized tissue and bone trabecula was defined by an increase in *COL1A1* and *COL10A1* (stage 3 vs. 4: *p* = 0.047) positive area. Stage 4 showed typical trabecular bone tissue. The relative volume of calcified tissue (mineralized cartilage and bone tissue) decreased with stages (stages 1-2 vs. 3: *p* = 0.002; stage 1-2 vs. 4: *p* = 0.012). *COL2A1* expression and stained area decreased from stages 1-2 to 4 (*p* = 0.010 and *p* = 0.004). *ACAN* expression decreased from stage 1-2 to stage 3 (*p* = 0.049) and stage 4 (*p* = 0.002).

**Conclusion:**

Loose bodies show tissue characteristics similar to endochondral ossification. They are probably a relevant substrate for regenerative therapeutic interventions in joint disease.

## Introduction

Loose bodies are free-floating (osteo-)chondral tissue fragments found in the synovial tissues or joint space of the knee, elbow, or shoulder.^1^ Several synonyms are used to describe loose bodies, including corpora libra,^2^ joint mice,^1^ rice bodies,^3,4^ and subcutaneous bodies. Loose bodies are small tissues of a few millimeter to centimeter in size and can be detected in magnetic resonance imaging, x-ray and CT scans.^5,6^ Asymptomatic loose bodies can reside for a long time in the joints before being diagnosed. Symptomatic loose bodies can cause pain, swelling, intermittent locking, dysfunction, or reduced mobility of the joint, which then lead to the need of surgical removal of the loose body.^1,6^ It is likely that more than one loose body is found in an affected joint of patients.^2^

Macroscopically, loose bodies are characterized by a cartilage superficial layer and a bony core.^1^ On microscopic level, they are characterized by a 3-zonal organization. The outer superficial zone is formed of flattened fibroblast-like cells and a more fibrous matrix, which passes into a transitional zone (highest collagen content), with a mixed population of round chondrocyte-like cells. The high abundance of proteoglycans is characteristic for the inner radial zone. Melrose described also a necrotic core in the center of the tissue.^2,7^ A high cell density is also characteristic for the loose bodies that are rich in proteoglycans.^7^

The origin of loose bodies is controversially discussed: They can form as a secondary product of degenerative joint diseases (e.g., osteoarthritis [OA]) or derive from detached (micro-)fragments in patients with osteochondritis dissecans (stage IV of the International Cartilage Regeneration & Joint Preservation Society), tissue fragments after fracture (e.g., flake fracture), a torn piece of cartilage after injury, or synovial chondromatosis.^2,6-8^ The loose bodies that we investigated in this study are of larger size (millimeter to centimeter scale) and were found in lower numbers in OA patients during endoprosthesis implantation compared to the high number of bodies in patients with chondromatosis. The characteristic small bodies found in patients with synovial chondromatosis, which is mainly diagnosed in young adults, were not included in this study.^9,10^

The cartilaginous matrix loose bodies might be an alternative cell source for chondrocyte isolation. Loose bodies collected from patients with diagnosed osteochondritis dissecans (aged 17-50 years) have been proposed to be a promising cell source for autologous chondrocyte implantation. Chondrocytes isolated from loose bodies and chondrocytes isolated from macroscopically intact articular cartilage showed a high capacity for long-term *in vitro* expansion and a better redifferentiation capacity (higher *ACAN, COL2*, and reduced *COL10* and *MMP13* levels) compared to bone marrow–derived mesenchymal stromal cells.^2^ Isolating these chondrocytes would even reduce donor site morbidities as the loose bodies are removed during knee surgery. Some of these loose bodies comprise articular cartilage-like tissue. Therefore, loose bodies might be used as a model to understand mechanisms related to how hyaline cartilage can be formed in adult people. One approach could be to culture the cartilaginous parts of loose bodies *ex vivo* with or without chondrogenic factors (e.g., growth factors) and investigate changes on cell phenotype and/or extracellular matrix (ECM) deposition. On the other side, loose bodies are a potential model to study cartilage to bone transition as they often comprise both cartilaginous and bone tissue. Again, stimulating factors associated with chondrocyte hypertrophy or bone formation (e.g., bone morphogenic proteins, β-glycerophosphate, and inflammatory cytokines) could be added to the culture media during *in vitro* culture.

Based on the gross morphological appearance of loose bodies found within one joint, we hypothesize that loose bodies found in the joint capsule can be grouped in different stages with tissue characteristics similar to that of endochondral bone formation.

Endochondral bone formation describes the process of bone formation from a cartilage template. Mesenchymal stromal cells condensate, differentiate into proliferating chondrocytes, mature toward hypertrophic chondrocytes, and the cartilage is invaded by blood vessels and gradually replaced by bone tissue.^11,12^ In this study, we collected loose bodies from patients undergoing knee, hip, and shoulder replacement. Cartilage- and bone-specific markers were characterized using histology and gene expression analysis. Tissue morphology and µCT scans were used to group the loose bodies in 4 stages, representing different stages of tissue maturation with similar characteristics to endochondral ossification.

## Materials and Methods

### Tissue Collection

Loose bodies were harvested from patients (*n* = 30 patients, aged between 27 and 87 years, Suppl. Table S1) undergoing total knee, hip, or shoulder replacement after obtaining written consent. The harvest of tissue was approved by the institutional review board of the University Hospital Magdeburg (N86/20). The loose bodies were cut in quarters to investigate gene expression and histological stainings, as well as µCT from similar pieces of the loose body. Tissue pieces found in the joint space were considered as loose bodies when a fibrous tissue shell was formed around the tissue. Patients with diagnosed chondromatosis at the time of joint replacement surgery were excluded from this study.

### Micro-Computed Tomography

To quantify the bone volume by volume of calcified tissue within the loose bodies, samples were thawed to room temperature and scanned using the Phoenix Nanotom 180S micro CT (70 kV, 160 µA, 6 µm pixel size, and 750 ms exposure time; GE Sensing & Inspection Technologies GmbH). After 3D image reconstruction (phoenix dataosx 2 rec, Phoenix contact), µCT data was analyzed (CT Analyser Version 1.18.8.0, Bruker) to calculate total tissue volume (TV) and calcified tissue volume (CV). TV represents the total tissue volume (cartilage, bone, and soft tissue volume) of the loose body. CV represents the volume of calcified tissue (calcified cartilage and bone tissue) within the TV of each sample. To differentiate between CV and TV, two thresholds were set for each sample during binarization. One threshold was adjusted to best match the regions of calcified tissue with the original image (CV) and a second threshold was set to match the regions of the total tissue volume with the original image (TV). The volumes (CV and TV) were calculated separately and the percentage of CV was calculated by setting the TV to 100%.

### Histological Processing

Loose bodies were fixed in neutral buffered formalin (4%, Otto Fischar GmbH & Co KG), decalcified (Entkalker soft SOLVAGREEN, Carl Roth), dehydrated, and paraffin embedded. Sections of 4 µm were cut (Hyrax M55 Microtome, Zeiss), dried overnight at 37°C and stored at room temperature. Before staining, slides were deparaffinized with decreasing concentration of alcohol (Ethanol absolut, Fischbar).

The safranin-orange staining was used to visualize and differentiate proteoglycan-rich ECM from bone-like and fibrous tissue. Slides were stained with fast green (FCF, 0.1% w/v, 1 minute, Sigma), washed with acetic acid (1%, 30 seconds, Carl Roth), and incubated with Safranin-orange (2% w/v, 30 minutes, Applichem). After washing with Ethanol (96%), slides were dehydrated with ethanol (96% and absolute ethanol), followed by xylene (Carl Roth), cover slipped (Kanadabalsam, Carl Roth), dried at room temperature, and imaged (Axio Observer, Zeiss).

The sirius Red staining allowed to analyze the orientation of collagen fibrils. Slides were stained with Sirius Red (0.13% w/v, Direct Red 80 in saturated picric acid, 60 minutes, Sigma Aldrich), washed with hydrogen chloride (0.01 N, 2 minutes, Carl Roth), deionized water (5 minutes), dehydrated, and cover slipped. Slides were imaged using polarized light (DIC channel, 15° to 20°, Axio Observer, Zeiss).

For immunofluorescence stainings, slides were blocked (4% w/v bovine serum albumin in PBS, 1 hour at room temperature, AppliChem), followed by antigen retrieval optimized for each antibody (see Suppl. Table S2). Slides were incubated with primary antibody (collagen I, II, and X [*COL1A1, COL2A1*, and *COL10A1*], *SOX9*, and *MMP13*) at 4°C overnight. After washing with washing buffer (TBST 1x, ChemCruz, supplemented with 0.05% Tween 20, AppliChem), secondary antibody (Alexa Fluor 555 donkey anti rabbit, 2 mg/ml, Invitrogen A31572) was incubated for 1 hour at room temperature. Respective isotype controls were used for all primary antibodies at the same concentration. After washing, slides were cover slipped (Roti-Mount Fluorcare DAPI) and dried at 4°C protected from light. Slides were imaged using a fluorescence microscope (Axio Observer, Zeiss).

### Quantitative Image Analysis of Immunofluorescent Stained

For the quantification of the positive stained area (COL1A1, COL2A1, and COL10A1), four images per loose body and antibody were used. Images were acquired with the same settings and magnification, and the threshold was manually adapted and kept the same for all donors (threshold COL1A1: 20, COL2A1: 10, and COL10A1: 15). Positive area and total tissue area were manually selected (ImageJ, U. S. National Institutes of Health, USA) and percentage of positive area was calculated.

### Gene Expression Analysis

Loose bodies were transferred in RNA stabilizing reagent (RNA later, Ambion), snap frozen. After thawing, RNA later was removed and tissue was pulverized in liquid nitrogen, and lysed with Trizol (Invitrogen) following manufacturer’s instruction for total RNA isolation. RNA was loaded on Nanoquant plate (TECAN Deutschland) to determine RNA concentration (Infinite F200 Pro, TECAN Deutschland. At total of 1 µg RNA was reverse-transcribed using high-capacity cDNA reverse transcription Kit (Thermo Scientific) and T100 Thermal cycler (BioRad) following manufacturer’s instruction. Quantitative realtime PCR was carried out on Quant Studio 6 Flex (Applied Biosystems, Thermo Fischer Scientific) using SYBR Green PCR Master Mix (ABI life technologies). All samples were analyzed using a selection of genes (*ACAN*, *COL1A1, COL2A1, COL10A1*, *FN*, *SOX9*, *MMP13*, 10 µM final concentration; Suppl. Table S3). Standard curves were used for absolute quantification of target genes and normalized to the reference gene (glyceraldehyde 3-phosphate dehydrogenase [GAPDH]).

### Statistical Analysis

Statistical analysis was performed using GraphPad Prism (GraphPad Software V8.3.0). In addition, 25% and 75% percentile with a line at the median are plotted. All data sets were tested for normality using the Shapiro-Wilk normality test. All data were tested for statistical significance using either nonparametric Kruskal-Wallis test with Dunn’s multiple comparisons test or one-way analysis of variance (ANOVA) with Holm-Sidak *post hoc* test to correct for multiple comparisons. A *p* value <0.05 was considered statistically significant.

## Results

### Morphological Appearance of Loose Bodies

**Figure 1.**
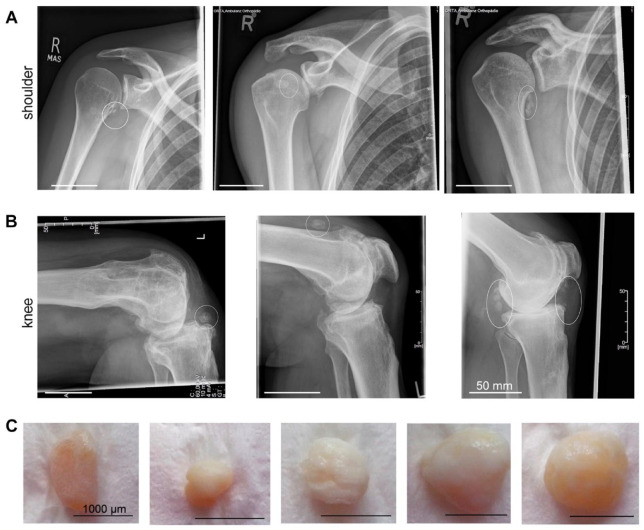
Morphological appearance of loose bodies. (**A, B**) Representative anterior-posterior of the shoulder and the knee joint of patients with loose bodies (highlighted with white circle). Loose bodies are found at different locations within the intra-articular space. Scale bar: 50 mm. (**C**) Macroscopic image of loose bodies after harvesting from patient tissue show an ellipsoidal- to oval-shaped tissue with a white, regular cartilage surface. The bony core is visible through the translucent cartilage cover in some of the loose bodies. Scale bar: 1,000 µm.

Loose bodies were visible in the radiographs of patients (**Fig. 1A** and **B**) due to their large size and tissue mineralization. The size of the loose bodies varied in between hundreds of micrometers to centimeter (**Fig. 1C**). Macroscopically, the loose bodies showed an ellipsoidal to round shape with cartilaginous-like white tissue on the surface of a more bone-like center. A thin layer of fibrous tissue was covering the surface.

### Loose Bodies Are Grouped in 4 Groups and Resemble Different Stages Similar to Endochondral Bone Formation

**Figure 2.**
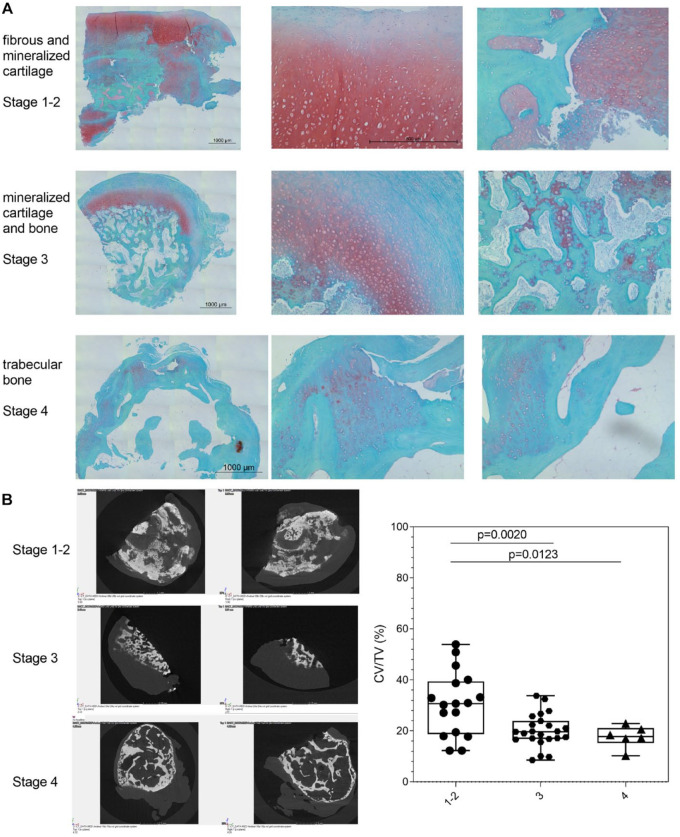
Four developmental stages of loose bodies resemble endochondral bone formation. (**A**) Safranin-orange staining of the cross section of the tissue at different stages. Scale bar: 1,000 µm. On the right side, higher magnification of safranin-orange images is shown. Scale bar: 500 µm with same magnification in respective column. (**B**) µCT images of representative samples of stages 1-2, 3 and 4, and quantification of CV relative to the TV. Scale bar: 1.0 mm. One-way ANOVA with Sidak *post hoc* test. ANOVA summary: number of treatments: 3, total number of values: *n* = 46, F = 8.250, *p* = 0.0009. CV = calcified tissue volume; TV = total tissue volume; ANOVA = analysis of variance.

Safranin-orange-stained sections and µCT data were used to group samples in 4 maturation stages using the criteria defined in Suppl. Table S4. We identified the following four stages: (1) fibrous, (2) (mineralized) cartilaginous, (3) cartilage and bone, and (4) bone with its typical trabecula structure. The samples collected in this study showed a stage-specific tissue composition, collagen organization, and gene expression pattern. A fibrous-like tissue layer was covering the surface of all loose bodies. Proteoglycan-rich areas were present in loose bodies of stage 1 to 4 (**Fig. 2A**). This interface with tight contact of the fibrous tissue to cartilaginous and mineralized tissue or bone trabecula was observed in the samples irrespective of their composition and stage. Samples of stage 1 mainly comprise fibrous tissue with limited proteoglycans mainly present in the pericellular matrix of the round chondrocyte-like cells. Areas of proteoglycan-rich cartilaginous tissue with chondrocyte-like cells were characteristic for samples in stages 2 and 3. Some samples showed a tree-bark-like appearance with alternating rows of cartilaginous, mineralized, and hypertrophic areas. First signs of immature bone tissue formation were present in stage 2. In these areas, the proteoglycan-rich areas seem to be replaced with mineralized tissue. In stage 3, areas of a rather mature bone tissue were present with first signs of bone trabeculae. At stage 4, loose bodies showed mature bone tissue with bone marrow–like structures filling the cavities between the bone trabeculae.

The fibrous surface layer contained a high density of spindle-shaped fibroblast-like cells. A high cell density of round chondrocyte-like cells was present in the cartilaginous areas of stages 1 to 3. Loose bodies at stage 2 and stage 3 showed a more organized cell orientation in column structures in the cartilaginous area. Larger areas of cartilage-like tissue were found in stages 2 and 3 with more chondrocyte-like cells. Immature bone tissue and newly formed trabecula (stage 3) showed a higher density of bone cells compared with the cell density in mature bone tissue in stage 4.

The µCT scans showed mineralized tissue from stage 1 - 2 on (**Fig. 2B**). In stages 1 - 2, some areas of the fibrous and cartilaginous tissue undergo mineralization, characterized by a dense mineralized tissue with limited porosity. The total CV relative to the TV showed the highest value in stages 1 - 2 (median: 30.66, *p* = 0.0020 and *p* = 0.0123 compared with stage 3 and stage 4). In stage 3, calcified tissue was present next to areas of tissue undergoing bone transition and immature bone with a CV of 19.63% (*p* = 0.9810 compared with stage 4). Mature bone-like tissue with its typical trabecular structure was characteristic for stage 4 (17.76%).

### Loose Bodies Classified in the four Groups Showed a Stage-Dependent Collagen Profile

**Figure 3.**
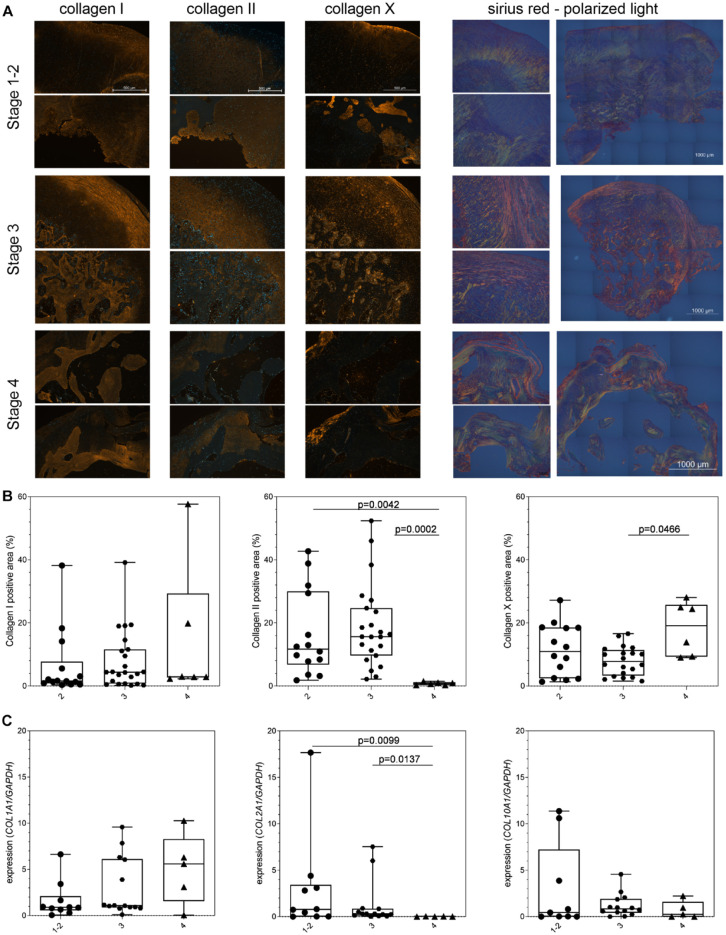
Collagen profile corroborates endochondral bone formation-like differentiation of loose bodies. (**A**) Immunofluorescence stainings for collagen I, II, and X. Scale bar 500 µm with same magnification in respective column. Polarized light images of Sirius-red-stained sections. Scale bar 1,000 µm. (**B**) Quantification of positive-stained area relative to total area. (**C**) Gene expression analysis for *COL1A1, COL2A1*, and *COL10A1*. Kruskal-Wallis test with Dunn’s multiple comparison test. Kruskal-Wallis summary: number of treatments 3, *p* values are 0.3077 (collagen I positive area, *n* = 43), 0.0004 (collagen II positive area, *n* = 43), and 0.0505 (collagen X positive area, *n* = 40); *p* values of gene expression analysis area are 0.2501 (*COL1A1,* *n* = 29), 0.0074 (*COL2A1*, *n* = 29), and 0.5419 (*COL10A1*, *n* = 28). *GAPDH* = glyceraldehyde 3-phosphate dehydrogenase; *COL1A1, COL2A1*, and *COL10A1* = collagen I, II, and X.

The organization and orientation of the collagen fibers showed differences in the different zones and stages (polarized light images of Sirius-red-stained sections in **Fig. 3A**). Throughout all stages, the fibrous layer showed circumferentially and parallel-aligned collagen fibers. Collagen fibers of this layer were sometimes bridging the tight interface to the underlying cartilaginous tissue. Areas of proteoglycan-rich cartilage showed collagen fibers perpendicular to the surface. Some of the samples showed an orientation resembling Benninghoff arcades of the collagen fibers typical for articular-like cartilage tissue (stages 2 and 3). Collagen fiber organization tended to increase in the cartilaginous areas from stage 1 to stage 3. A higher organization of the parallel-aligned collagen fibers was also characteristic for mature bone trabecula in stage 4 compared to the immature bony tissue in stage 3.

To discriminate between the collagen types in the ECM of the loose bodies, immunofluorescence stainings of the 3 collagen types (collagen I, II and X) were performed (**Fig. 3A**; IgG controls are shown in Suppl. Fig. S1). Collagen I was present in the fibrous shell regardless of the stage. The ECM in areas in close proximity to the transition area of cartilage-to-bone and next to immature bone tissue was positive for collagen I in stages 1 to 2 (median: 1.58%). Most collagen I positive area was found in stage 3 (median: 4.42%) with a slightly lower value in stage 4 (median: 2.94%, **Fig. 3B**). The ECM of immature and mature bone tissue (stages 3 and 4) was also positive for collagen I. No statistical significances were observed comparing the collagen I positive-stained areas of the four stages. The collagen II positive area decreased from stages 1 - 2 (median: 11.73%, *p* = 0.0042 compared with stage 4) over stage 3 (median: 15.62%, *p* = 0.0002 compared with stage 4) to stage 4 (median: 0.80). Collagen II was present in the pericellular region or the ECM at stages 1 to 3. Collagen II positive area was similar to the proteoglycan-rich areas identified in the Safranin-orange staining. This cartilaginous tissue showed no or limited staining for collagen I. At stage 4, no or very limited collagen II staining was observed. The fibrous lining layer on the surface of the samples was not positive-stained for collagen II.

The more hypertrophic-associated matrix protein collagen X was mainly present in stage 4 (median: 19.15%, *p* = 0.0466 compared with stage 3), followed by stages 1 - 2 (median: 10.96%) and lowest in stage 3 (median: 7.78%). The ECM of the trabecula bone tissue and bone marrow like tissue within the bone cavities also showed a positive collagen X staining. The respective isotype controls did not show a staining (Suppl. Fig. S1).

On gene expression level (**Fig. 3C**), *COL1A1* showed an increase in expression from stages 1 and 2 (median: 0.891) to stage 3 (median: 1.080) and reached a maximum at stage 4 (median: 5.594). No statistical differences were observed between groups. *COL2A1* was highest expressed in stages 1 and 2 (median: 0.781, *P* = 0.0099 compared with stage 4), followed by stage 3 (median: 0.306, *P* = 0.0137 compared with stage 4) and stage 4 (median: 0.002). *COL10A1* ranged between 0.448 and 0.8411, with no statistical differences between groups.

**Figure 4.**
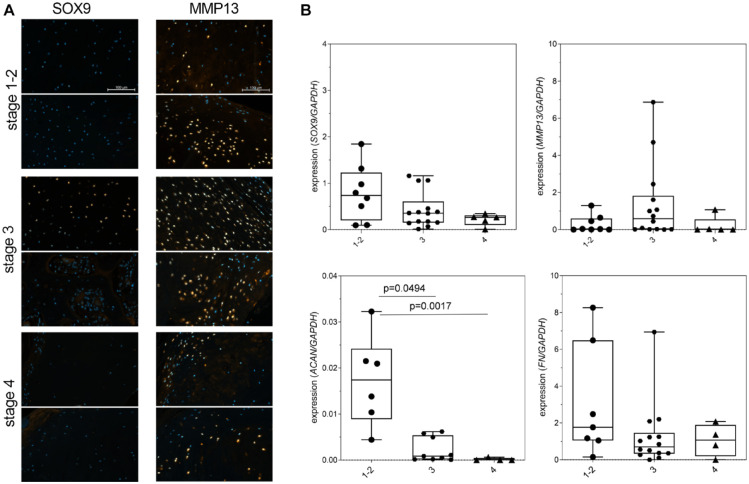
Changes in the chondrocyte phenotype during loose body differentiation. (**A**) Immunofluorescence stainings of SOX9 and MMP13. Scale bar: 100 µm with same magnification in each column. (**B**) Gene expression analysis for *SOX9, MMP13, ACAN*, and *FN*. Kruskal-Wallis test with Dunn’s multiple comparison test. Kruskal-Wallis summary: number of treatments 3, *P* values are 0.1493 (*SOX9, n* = 27), 0.0818 (*MMP13, n* = 27), 0.0001 (*ACAN, n* = 19), and 0.2368 (*FN, n* = 25). *MMP13* = matrix metalloproteinase 13; *ACAN* = aggrecan; *FN* = fibronectin.

Cells in the areas of cartilage-like tissue and cells with Safranin-orange positive pericellular matrix were stained for the chondrogenic transcription factor SOX9 (stages 1 to 3, **Fig. 4A**). Cell nuclei were positive stained for MMP13 in stages 1 to 3 in areas with signs of cartilage to bone transition. In addition, some cells in the fibrous capsule were positive-stained for MMP13. The transition from a more cartilaginous phenotype in stages 1 - 2 to stage 4 was accompanied by a decrease of *ACAN* (*p* = 0.0494 between stages 1 - 2, *p* = 0.0017 between 1-2 and 4 and 3 and *p* = 0.3326 between stages 3 and 4) and *SOX9* (no significant differences between groups) expression. There was no clear trend for *FN* and *MMP13* expression (**Fig. 4B**).

## Discussion

Loose bodies are often found in the knee, hip, or shoulder of patients with (degenerative) joint disease or after injury or fracture. These bodies could serve as an alternative tissue source to isolate chondrocytes for *in vitro* expansion and tissue engineering approaches.^2^ Whether loose bodies undergo maturation and can form cartilage and/or bone tissue is currently not fully understood. In this study, we hypothesized that loose bodies can be grouped in different stages with tissue characteristics similar to endochondral ossification.

Loose bodies showed characteristics of articular cartilage-like tissue, the transition from cartilage to bone or bone tissue. Based on these tissue characteristics, we identified four stages: (1) fibrous, (2) (mineralized) cartilaginous, (3) cartilage and bone, and (4) bone tissue. In previous studies, the outer, rather fibrous layer was referred to as superficial zone of the 3 to 4 zones (superficial, radial/transition, and inner zone) in loose bodies.^2,7^ The absence of proteoglycans and *COL2A1* in this fibrous layer suggests that this zone might not be of cartilaginous nature. However, this layer showed a tight connection to the underlying zones of the loose bodies, with collagen fibers bridging this interface (stages 2 and 3). It is also possible that this fibrous layer formed around the osteochondral fragments to minimize friction or serves as nutrition source for the cells within the loose body by connecting some of them to the blood supply. The presence of vascular-like structures in the fibrous layer does support this (Suppl. Fig. S2). In samples of stage 4, the intertrabecular space was filled with a soft tissue, sometimes similar to bone marrow.

Benninghoff arcades of collagen fibrils were formed in the cartilage zone rich in proteoglycans. The presence of these arch-like collagen orientation indicates the hyaline character of the cartilage tissue in the loose bodies.^13^ The Benninghoff arcades might have formed in the floating samples without the complex mechanical loading characteristic for the hyaline cartilage in the knee joint. Areas of hypertrophic cartilage (large chondrocytes residing in a matrix positive for collagen X and MMP13) and mineralized tissue were often alternating, leading to the typical tree-bark-like structure (stages 2 and 3). This zonality is similar to the zonal structure described by Textor *et al*.^2^ and Melrose.^7^ Whereas they reported of a bone core in the center of the loose bodies, we observed repeated areas of cartilage, hypertrophic and mineralized in the loose bodies.

The tissue characteristics of the loose bodies in the different groups and zones are similar to the developmental process of bone formation through endochondral ossification. The tendency of a higher expression of *COL2A1* and *ACAN* in stages 1 - 2 are typical markers for mesenchymal condensation and proliferative chondrocytes during early stage of endochondral ossification. *COLX* and *MMP13* are two markers present in the (pre-)hypertrophic zone of the growth plate. Chondrocytes in the hypertrophic zone stop proliferating and become larger in volume.^11,12^ The expression of *COL10A1* and *MMP13* in the different stages of the loose bodies did not significantly change in the samples included in this study. On protein level, an increase in collagen X positive area was characteristic for loose bodies in stage 4 compared with stage 3, the transition from cartilage and bone tissue characteristics to mature bone-like tissue. Therefore, it is assumed that the chondrocyte residing in the loose bodies differentiate toward a hypertrophic phenotype and mature toward bone-like tissue. This is supported by the finding that in stage 3 multiple areas of cartilaginous, hypertrophic/mineralized cartilage and immature bone tissue is present. This is in line with literature on the expression of *COLX* by hypertrophic chondrocytes residing in the hypertrophic zone.^14,15^ The positive staining for collagen X in the outer fibrous tissue surrounding the loose bodies, however, this was independent of the differentiation stage. The variation in tissue morphology and structure within a single loose body leads to a heterogeneous cell population. Thus, the gene expression data represent the average expression levels of this heterogeneous cell population and can explain the high variation of gene expression in each group. Analyzing the cell phenotype on single-cell level is challenging on mineralized tissue and is suggested to be investigated in further studies. The development of bone tissue from a cartilage template in the loose bodies is suggested by the fact that, in some loose bodies (stages 2 and 3), small areas with cartilage-like tissue were visible in regions where bone was forming and sometimes even within the trabecula. Whether all loose bodies or only a subgroup of these originating from a distinct origin can actually undergo the transition from a cartilaginous to a bone-like tissue remains an open discussion point of future studies. So far, studies on *in vitro*–engineered cartilage-like tissue from chondrocytes implanted *in vivo* show limited capacity to undergo endochondral bone formation.^16^ To which extent *in situ*–formed loose bodies with cartilaginous tissue can undergo the transition toward bone tissue *in vivo* needs to be investigated in future studies.

The different tissue characteristics of stages 1 to 4 can derive from the way the loose bodies are formed. Furthermore, the time the loose bodies resided or matured within the joint space could have an impact on the overserved stages. However, there is no clear evidence whether all loose bodies can mature while residing in the intra-articular joint space or whether this is limited to loose bodies coming from a distinct origin. The mechanisms controlling the composition and the origin of loose bodies are controversially discussed.^2,6-8^ Melrose^7^ correlated the tissue characteristics of the loose bodies to their origin/development: Fibrinous bodies result from intra-articular bleeding, cartilaginous loose bodies are caused by trauma in osteoarthritic joints, and osteocartilaginous tissues are caused by fractures or osteochondritis dissecans or synovial chondromatosis. This is not in line with our finding that loose bodies harvested from one patient with multiple loose bodies are often grouped in two different stages. We assume that, if multiple loose bodies are found in one patient, the origin of these loose bodies is likely to be the same. The finding of loose bodies showing different tissue characteristics (grouped in two different stages) indicates that the tissues might undergo a maturation or remodeling process over time. However, it remains unknown what factors stimulate the cell phenotype and the formation of the ECM of the loose bodies. So far, loose bodies of different origins were characterized by their bony core and cartilaginous shell.^1,2,6,7^ Interestingly, the stage 4 of loose bodies described in our study has not been described in literature before. The different origin and/or different duration of the loose bodies residing in the joint space could affect the cell phenotype, cell differentiation, and tissue remodeling. However, this is a challenging research question to be addressed.

The morphological characterization and the tissue composition of the loose bodies in this study lead to the assumption that the human body is able to form hyaline-like cartilage tissue in adults. The microscopic structure of the loose bodies characterized in this study distinguishes from osteochondral fragments detached due to trauma or osteochondritis dissecans: Osteochondral fragments consist of a cartilage layer with underlying subchondral bone with cartilage being present at only one side of the fragment.^1^ In contrast, the loose bodies in our study showed a core-shell or often a tree-bark-like structure with an ellipsoidal or round geometry. To form a similar loose body from a torn piece of (osteo-)chondral tissue, cartilage-like tissue needs to be formed in these tissues. Furthermore, the cartilage-bone interface of loose bodies showed a smooth transition between both tissues. The tree-bark-like structure and the multiple areas of cartilaginous, mineralized, hypertrophic, and bone tissue support the idea that loose bodies undergo a remodeling and/or maturation process rather than being a static or fully developed tissue. Whether all loose bodies or only a subgroup of these (e.g., different origin, age) undergo all stages, remains an open question.

The unclear origin, the time point, and duration the loose bodies are formed and reside in the joint space are one limitation of this study. Furthermore, the results of the loose bodies’ analyses shown in this study are a snapshot of the free-floating tissue specimens without knowing their real origin of formation in first place.

The observation of a fibrous-like tissue shell formed around the loose bodies indicated that the tissue was present for some time in the joint. A recently torn piece of cartilage (flake fracture) or osteochondral defect would have a less round and less smooth surface. An osteochondral fragment could be identified by the presence of the tidemark separating cartilage and bone. Irrespective of the origin of the loose bodies, one of the characteristics of loose bodies is the core shell structure. This characteristic is different from a chondral/osteochondral fragment and osteophyte and thus supporting the hypothesis that the loose bodies may undergo maturation.

To conclude, this study shows that loose bodies found in the joint can be grouped in stages similar to endochondral bone formation. Whether the loose bodies can actually undergo a maturation process while residing in the intra-articular joint space or whether the origin of the free-floating tissues is determining its stage as suggested in previous studies^2,7^ remains unknown. The four stages identified in this cohort of patients represented the different stages from fibrous, over cartilaginous, and mineralized tissue to bone trabecula formation. Assuming that loose bodies can undergo maturation processes, the tissue characteristics of the loose bodies indicated that it is possible to form articular cartilage tissue in adults. The intra-articular environment seems to serve as a natural bioreactor to stimulate chondrogenesis, mineralization, and ossification in the floating tissues, depending on the stage of the loose body. Loose bodies might also be used as a model to study the impact of an inflammatory and/or degenerative environment on cartilage-bone tissues. Furthermore, loose bodies could probably be a relevant substrate for regenerative therapeutic interventions in joint diseases.

## Supplemental Material

sj-docx-1-car-10.1177_19476035231212608 – Supplemental material for Loose Bodies Found in the Human Intra-Articular Space Showed Characteristics Similar to Endochondral Bone Formation Supplemental material, sj-docx-1-car-10.1177_19476035231212608 for Loose Bodies Found in the Human Intra-Articular Space Showed Characteristics Similar to Endochondral Bone Formation by Andrea Schwab, Thomas Pap, Veit Krenn, Wolfgang Rüther, Christoph Lohmann and Jessica Bertrand in CARTILAGE
