# Supplementary material for: Loose Bodies Found in the Human Intra-Articular Space Showed Characteristics Similar to Endochondral Bone Formation
Source: Cartilage. 2023 Dec 1;15(4):353–62. doi: 10.1177/19476035231212608 (PMC11519999; doi:10.1177/19476035231212608)
Supplement: sj-docx-1-car-10.1177_19476035231212608 – Supplemental material for Loose Bodies Found in the Human Intra-Articular Space Showed Characteristics Similar to Endochondral Bone Formation [file sj-docx-1-car-10.1177_19476035231212608.docx]

**Supplementary information**

Table S1: Overview of patient data loose bodies were harvested from.

| **Loose body number** | **Sex** | **Age** | **Location** |
| --- | --- | --- | --- |
| 1 | male | 70 | knee |
| 2 | female | 27 | shoulder |
| 4 | female | 71 | knee |
| 5 | female | 83 | knee |
| 6 | male | 24 | shoulder |
| 7 | male | 77 | knee |
| 8 | male | 72 | knee |
| 9 | male | 57 | knee |
| 10 | female | 66 | knee |
| 11 | male | 75 | knee |
| 12 | male | 81 | hip |
| 13 | female | 76 | knee |
| 14 | female | 57 | knee |
| 15 | female | 77 | knee |
| 16 | female | 62 | knee |
| 17 | female | 50 | knee |
| 18 | female | 45 | knee |
| 19 | male | 63 | knee |
| 20 | male | 62 | knee |
| 21 | male | 75 | knee |
| 23 | male | 65 | knee |
| 24 | female | 87 | knee |
| 26 | male | 73 | knee |
| 27 | male | 78 | knee |
| 30 | female | 52 | knee |
| 31 | Male | 70 | knee |
| 32 | male | 54 | knee |
| 33 | female | 62 | knee |
| 34 | male | 60 | knee |
| 35 | male | 30 | shoulder |

Table S2: Overview of primary antibodies for immune fluorescence stainings.

| **Antibody** | **Concentration** | **Catalogue number** | **Producer** | **Antigen retrieval** | **Time** |
| --- | --- | --- | --- | --- | --- |
| **Collagen type I** | 1 mg/ml  use: 1:500 | NB600-408 | NovusBio | Hyaluronidase (4mg/ml in PBS pH 5,5) | 15 min |
| **Collagen type II** | 200 µg/ml  use 1:200 | sc-28887 | Santa Cruz | Hyaluronidase (10mg/ml in PBS) | 30 min |
| **Collagen type X** | 1 mg/ml  use 1:500 | ab58632 | Abcam | Trypsin (0.05% in water) | 10 min |
| **SOX9** | 1 mg/ml  use 1:500 | AB5535 | Merck Millipore | Trypsin (0.05% in water) | 10 min |
| **MMP13** | 1 mg/ml  use 1:500 | ab39012 | Abcam | Pronase (1mg/ml in PBS) | 30 min |

Table S3: Overview of primer (gene name, forward and reverse sequence) used for gene expression analysis.

| **Gene** | **Sequence forward** | **Sequence reverse** |
| --- | --- | --- |
| ***GAPDH*** | CCC ACT CCT CCA CCT TTG AC | AGC CAA ATT CGT TGT CAT ACC AG |
| ***COL1A1*** | AGG GCC AAG ACG AAG ACA TC | ATC ACG TCA TCG CAC AAC AC |
| ***COL2A1*** | CTG GAA AGC CTG GTG ATG ATG GTG | TGT GAC CTT TGA CAC CAG GAA GG |
| ***COL10A1*** | AAT CCC TGG ACC GGC TGG AAT TTC | TTG ATG CCT GGC TGT CCT GGA ACC |
| ***FN*** | CCA CCC CCA TAA GGC ATA GG | GTA GGG GTC AAA GCA CGA GTC ATC |
| ***SOX9*** | GAG AGC GAG GAG GAC AAG TTC | TCG CTC TCG TTC AGA AGT CTC |
| ***MMP13*** | CTT GAC CAC TCC AAG GAC CC | GCG CCA GAA GAA TCT GTC TTT |
| ***OPN*** | TCC TAG CCC CAC AGA CCC TT | ATA ACT GTC CTT CCC ACG GC |
| ***ACAN*** | CTC CGG AAT GGA AAC GTG AAT C | CTG GTA GTC TTG GGC ATT GTT G |

Table S4: Characteristics of the identified stages of loose bodies undergoing a process similar to endochondral ossification.

|  | Stage 1-2 | Stage 3 | Stage 4 |
| --- | --- | --- | --- |
|  | **Fibrous and (mineralized) cartilage** | **Cartilage and bone tissue** | **Bone tissue with trabecula** |
| Safranin-O staining | Fibrous shell | Fibrous shell | Fibrous shell |
|  | Proteoglycan rich cartilage like tissue | Proteoglycan rich cartilage like tissue | Limited proteoglycans in ECM |
| µCT | Tissue calcification | Tissue calcification and bone trabecula | Trabecular bone |
| Polarized light/ spatial organization | Mainly random orientation of collagen fibers | Collagens often formed Benninghoff arcades | Highly organized parallel fibrils |


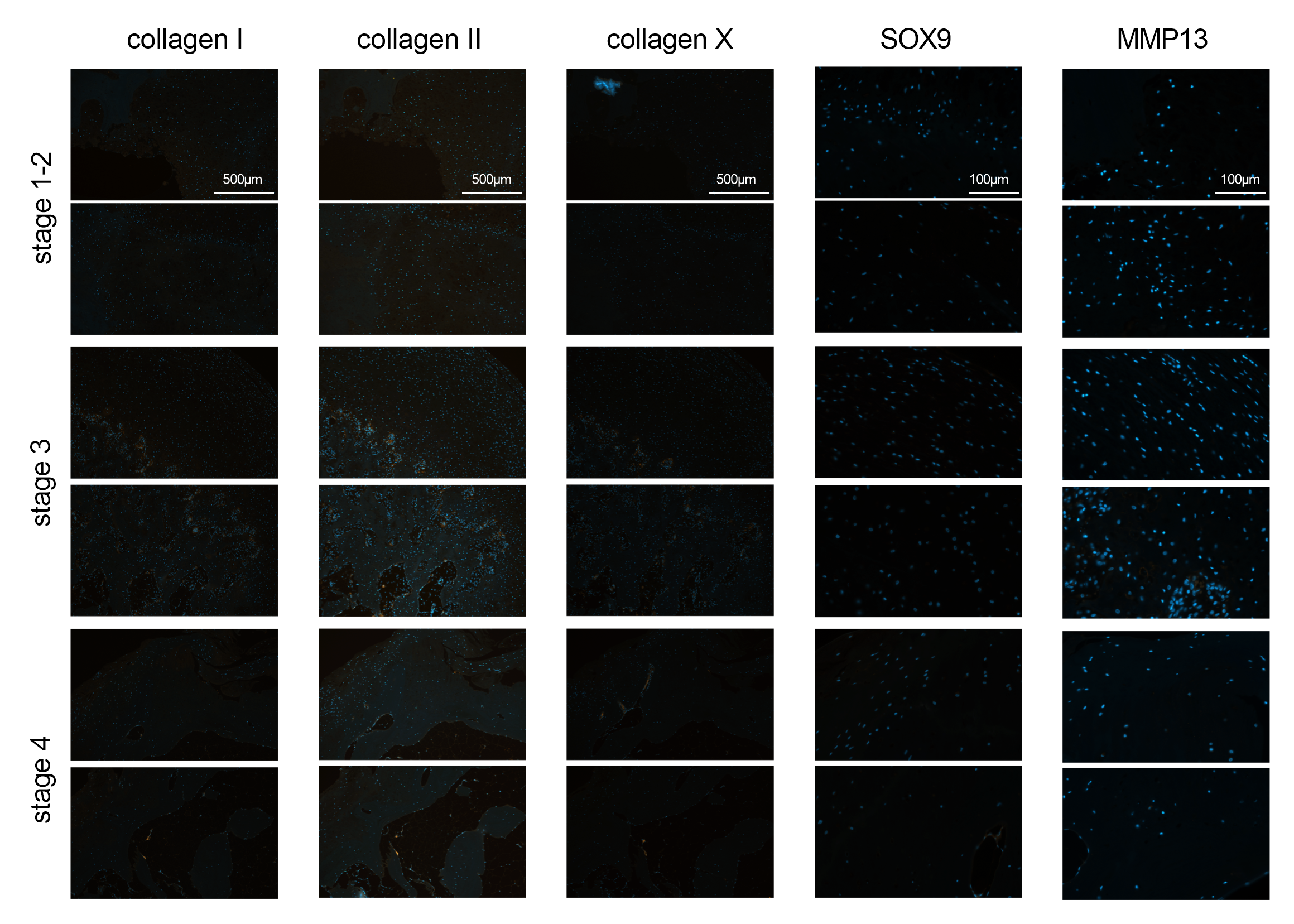


Figure S1: Isotype controls of immune fluorescence stainings for collagen type I, II, X0, SOX9 and MMP. Images were acquired at same magnification and exposure as primary antibodies in figure 3 and 4. Scale bar 500µm (collagen I, II, X) and 100µm (SOX9, MMP13).


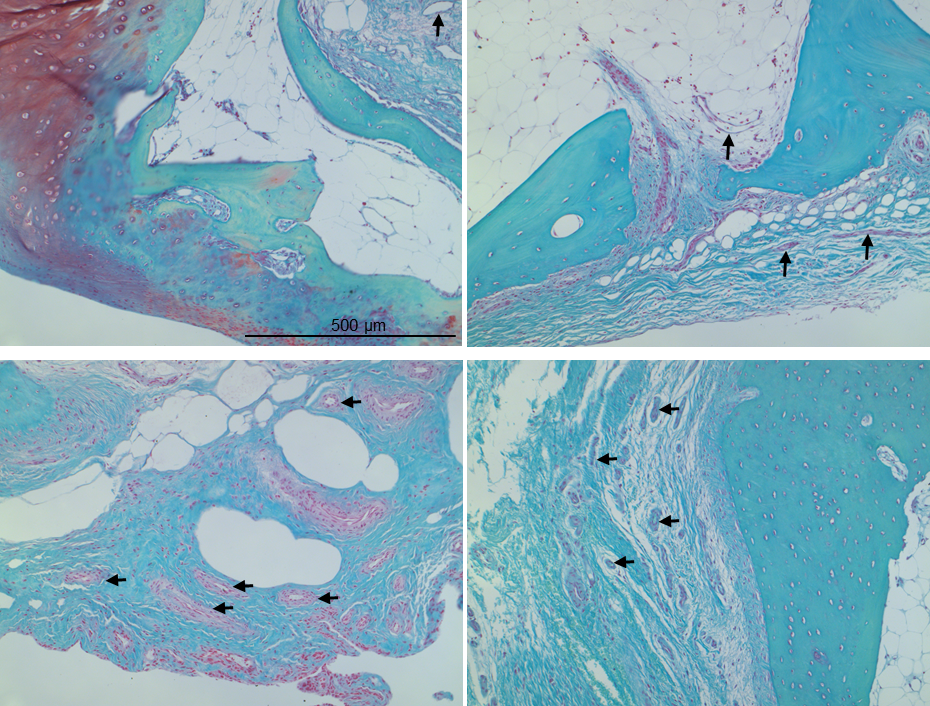


Figure S2: Safranin-orange images highlighting blood vessels (indicated by black arrows) in the fibrous shell and inside the loose bodies. Scale bar 500 µm.
